# Chronological Reorganization of Microtubules, Actin Microfilaments, and Chromatin during the First Cell Cycle in Swamp Buffalo (*Bubalus bubalis*) Embryos

**DOI:** 10.4061/2010/382989

**Published:** 2010-12-22

**Authors:** Vibuntita Chankitisakul, Theerawat Tharasanit, Kriengsak Tasripoo, Mongkol Techakumphu

**Affiliations:** ^1^Department of Obstetrics, Gynaecology and Reproduction, Faculty of Veterinary Science, Chulalongkorn University, Bangkok 10330, Thailand; ^2^Research and Development Center for Livestock Production Technology, Faculty of Veterinary Science, Chulalongkorn University, Bangkok 10330, Thailand

## Abstract

This paper aimed to study the dynamics of early embryonic development, in terms of redistribution of cytoskeleton (microtubules, actin microfilaments) and chromatin configurations during the first cell cycle in swamp buffalo embryos. Oocytes were matured and fertilized *in vitro*, and they were fixed at various time points after IVF. At 6 h after IVF, 44.4% matured oocytes were penetrated by spermatozoa. Partial ZP digestion, however, did not improve fertilization rate compared to control (*P* > .05). At 12 h after IVF, the fertilized oocytes progressed to the second meiotic division and formed the female pronucleus simultaneously with the paternal chromatin continued to decondense. A sperm aster was observed radiating from the base of the decondensing sperm head. At 18 h after IVF, most presumptive zygotes had reached the pronuclear stage. The sperm aster was concurrently enlarged to assist the migration and apposition of pronuclei. Cell cleavage was facilitated by microfilaments and firstly observed by 30 h after IVF. In conclusion, the cytoskeleton actively involves with the process of fertilization and cleavage in swamp buffalo oocytes. The centrosomal material is paternally inherited. Fertilization failure is predominantly caused by poor sperm penetration. However, partial digestion of ZP did not improve fertilization rate.

## 1. Introduction

Fertilization in mammals requires a successful series of events involving a profound remodeling of the nucleus and cytoplasm of both spermatozoa and oocytes. Microtubules and actin microfilaments have been demonstrated to dynamically play an important role during fertilization and cleavage in a number of species. The microtubules actively involve in the process of fertilization by the formation of microtubule networks that facilitate the migration and apposition of male and female pronuclei. These microtubules are paternally inherited in most mammalian species, including human [1, 2], sheep [3], rabbit [4], porcine [5], bovine [6–8], and rhesus monkey [9]. On the other hand, the paternal centrosome in the ooplasm is functionally absent in mice, and thus the syngamy of the two pronuclei requires the maternal centrosome [10, 11]. In addition, the evidence that a reversible microfilament depolymerizer (cytochalasin B) fails to inhibit the movement of male and female pronuclei but it adversely affects the syngamy and cell division [12] suggests an important role of actin microfilaments during cellular cleavage [3, 4]. However, these events on gamete interaction and early embryo development especially during fertilization have been poorly reported in the swamp buffalo. It has only been morphologically studied *in vivo* [13]. Understanding the redistribution patterns and role of microtubules and actin microfilaments during fertilization *in vitro *will provide fundamental knowledge of early embryo development and may improve *in vitro* embryo production techniques principally by the characterization of factors associated with fertilization failure in this species. The present research was designed to study the dynamics of early embryonic development, in terms of redistribution of cytoskeleton (microtubules, actin microfilaments) and chromatin configurations during the first cell cycle in swamp buffalo embryos.

## 2. Materials and Methods

### 2.1. Chemicals

All chemicals used in this study were purchased from Sigma-Aldrich Chemical Co. (St. Louis, MO, USA), unless otherwise stated.

### 2.2. In Vitro Maturation (IVM)

Swamp buffalo ovaries were obtained from animals of unknown reproductive status at a local slaughterhouse, then they were transported to the laboratory within 4 h in 0.9% (w/v) normal saline supplemented with 100 IU/mL penicillin G and 100 *μ*g/mL streptomycin at 28–35°C. The ovaries were washed once in 70% (v/v) alcohol and 0.9% (w/v) normal saline [14]. The oocytes were later aspirated from 2–8 mm antral follicles with an 18-gauge needle attached to a 10 ml syringe. The cumulus oocyte complexes were morphologically selected under a stereomicroscope at 400x magnifications. Cumulus-oocyte complexes (COCs) with homogenous ooplasm and surrounded by compact multiple layers of cumulus cells were submitted to *in vitro* maturation. Groups of 10 COCs were cultured in 50 *μ*L droplets of NaHCO_3_-buffered tissue culture medium 199 covered with mineral oil supplemented with 10% (v/v) buffalo follicular fluid, 50 IU/mL human chorionic gonadotropin (Intervet, Boxmeer, The Netherlands), 0.02 IU/mL follicle stimulating hormone, 1 *μ*g/mL estradiol-17*β*, 100 *μ*M cysteamine, 20 ng/mL epidermal growth factor, 100 IU/mL penicillin G, and 100 *μ*g/mL streptomycin. Three pools of follicular fluid were obtained from 2–8 mm follicles, then sterilized by filtering through the 0.22 *μ*m syringe driven filter, and then stored in sterile microcentrifuge tubes at −80°C. IVM was performed at 38.5°C for 22 h in a humidified atmosphere of 5% CO_2_ in air.

### 2.3. Partial Digestion of Zona Pellucida (ZP)

After *in vitro* maturation, oocytes were denuded and were transferred into 30 *μ*L droplet of an acid Tyrode's solution (pH 3.1) for 45 sec at room temperature (28–30°C). They were washed immediately two times with 2 ml of a modified Tyrode's (TALP) medium. ZP-digested oocytes were submitted to fertilization and culture procedures as mentioned above. Percentage of pronuclear formation was recorded at 18 h after IVF. Non-Tyrode treated oocytes served as control.

### 2.4. In Vitro Fertilization and In Vitro Culture (IVF and IVC)

Frozen semen from a fertile bull was used in this study. The semen was thawed at 37°C for 30 sec and then submitted to swim-up procedure for 45 min in TALP medium supplemented with 10 *μ*g/mL heparin as described by Parrish et al. [15]. Groups of 10–15 COCs in TALP medium, supplemented with 20 *μ*M penicillamine, 10 *μ*M hypotaurine, and 1 *μ*M epinephrine, were fertilized with sperm at a final concentration of 2 × 10^6^ sperm/mL [16]. IVF was performed at 38.5°C in a humidified atmosphere of 5% CO_2_, 5% O_2_ for 12 h. Excessive cumulus cells and sperms were then removed by repeated pipetting in culture medium containing 1 mg/mL hyaluronidase. Ten to fifteen presumptive zygotes were then cultured in 50 droplets of synthetic oviductal fluid containing 1% (v/v) fetal calf serum, 100 IU/mL penicillin G, and 100 *μ*g/mL streptomycin at 38.5°C in an atmosphere of 5% CO_2_, 5% O_2_.

The presumptive zygotes were randomly fixed and examined at 6, 12, 18, 24, and 30 h after IVF. Prior to fixation, they were incubated for 45 min at 37°C in a glycerol-based microtubule-stabilizing solution that contained 25% (v/v) glycerol, 50 mM MgCl_2_, 0.1 mM EDTA, 1 mM 2-mercaptoethanol, 50 mM imidazole, 4% Triton-X-100, and 25 *μ*M phenylmethylsulfonyl fluoride at pH 6.7 [17]. Subsequently, they were fixed and stored in 4% (w/v) paraformaldehyde in PBS until analysis.

### 2.5. Fluorescent Labeling of Oocytes and Presumptive Zygotes

To label the microtubules, the presumptive zygotes and embryos were first incubated at 25°C for 1 h in a 1 : 100 solution of monoclonal anti-*α*-tubulin (clone B1-5-1-2) in 0.1% (v/v) Triton-X-100 in PBS-BSA. They were subsequently washed in PBS-BSA and incubated for 1 h in a 1 : 100 solution of a goat antimouse second antibody conjugated with tetramethylrhodamine isothiocyanate (TRITC) in PBS-BSA. After washing twice in PBS-BSA, the actin microfilaments were stained by incubation for 30 min in a solution of 0.165 *μ*M Alexa Fluor 488 phalloidin (Molecular Probes, Invitrogen, OR, USA) in PBS-BSA. In addition, they were subsequently stained for 10 min with Alexa Fluor 594 wheat germ agglutinin (WGA; Molecular Probes) to locate lectin-rich ZP. Finally, the presumptive zygotes and embryos were incubated for 15 min with 20 *μ*M DAPI to label the chromatin. Labeled samples were mounted on a glass microscope slide in a 2 *μ*L droplet of antifade medium (Vectashield, Vector Lab, CA, USA) to retard photobleaching.

### 2.6. Confocal Laser Scanning Microscopy (CLSM)

Confocal laser scanning microscopy (C1, Nikon, Japan) was used to demonstrate the presence or absence of sperm within ooplasm (sperm penetration rate) at 6 h after IVF. Three laser sources from Diote 408 nm, Argon 488 nm, and HeNe 543 nm were used to simultaneously excite the fluorescent signals from DAPI, AlexaFluor 488 phalloidin (microfilaments), and Alexa Fluor 594 (ZP), respectively. The digital micrographs produced using the sequential scanning mode for the 3 separate colors were merged into single panel using EZ-C1 software (Nikon, Japan). The resulting multicolor micrographs were subsequently examined using Adobe Photoshop CS (Adobe System Inc., Mountain View, CA, USA).

### 2.7. Experimental Design

A total of 63 presumptive zygotes were examined at 6 h after IVF for sperm penetration using confocal laser scanning microscopy. Phalloidin and WGA were used to localize the boundary of the ooplasm and lectin-rich ZP in order to facilitate the visualization of sperm within the ooplasm, and DAPI was used to label the chromatin. The presence of a spermatozoon within ooplasm indicated the sperm penetration, and the oocytes having sperm head(s) bound onto or within the ZP were considered as nonfertilized oocytes.

To test whether partial digestion of ZP prior to IVF would improve the sperm penetration rate, thereby improving IVF efficiency, a total of 64 oocytes were treated with acid Tyrode's solution and then fertilized *in vitro*. Fertilization rate was assessed by percentage of male and female pronuclear formation at 18 h after IVF.

For the distribution pattern of the cell cytoskeleton and chromatin configurations during fertilization and early embryo development, 378 presumptive zygotes and/or embryos were fixed at 12, 18, 24, and 30 h after IVF. Following immunolabeling with monoclonal anti-*α*-tubulin-TRITC, Phalloidin, and DAPI to identify microtubules, microfilaments, and chromatin, respectively, they were examined using immunofluorescent microscopy (BX51, Olympus, Tokyo, Japan). The characteristics of the chromatin, polar body, cytoskeleton, sperm heads, and pronuclear formation were recorded. Oocyte activation was defined by the progression of chromosomal development after IVF from metaphase II (MII) through telophase II and also the formation of female pronucleus. Activated oocytes having decondensing sperm head and/or formation of male pronucleus and cell cleavage were identified as fertilized oocytes/zygotes. Metaphase I (MI), anaphase I, or telophase I oocytes were classified as nonmatured oocytes. The oocytes that had a dispersed pattern of chromatin and cytoskeleton were classified as degenerate oocytes.

### 2.8. Statistical Analysis

Descriptive data was used to describe the chronology of early embryo development in terms of the redistribution of cytoskeleton (microtubules and actin microfilaments) and chromatin configurations. Differences in percentage data of maturation and fertilization among stages were presented as mean and analyzed by Fisher's exact test (SAS 9.1, The SAS Institute Inc., Cary, NC, USA). *P*-values less than.05 were interpreted as significant.

## 3. Results

### 3.1. Sperm Penetration

**Figure 1:**
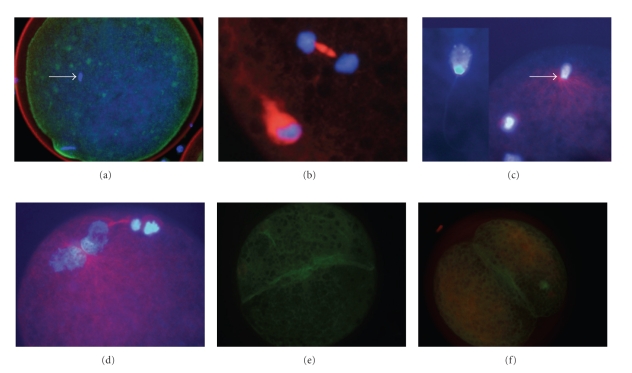
Images of swamp buffalo oocytes after staining with wheat-germ agglutinin (red) to localize the outline of the ZP, monoclonal-*α*-tubulin-TRIT C (red) to demonstrate microtubules, Alexa 488 phalloidin (green) to identify microfilaments, and DAPI (blue) to label the chromatin. (a) was illustrated using confocal microscopy. (b)–(f) were illustrated by immunofluorescent microscopy. An intact spermatozoon (arrow) was present within the cytoplasm of the MII oocytes at 6 h after IVF (a) At progression of chromosomal development after IVF from MII, the oocytes entered telophase II with one polar body, and the condensed chromatin were found at the pole of the astral microtubule (b). Sperm aster extended (arrow) from the base of the decondensed sperm head (c). Migration and apposition of male and female pronuclei were assisted by the dense array of microtubules found between pronuclei (d). Microfilaments were condensed in the middle line of the cell furrow during cleavage (e). A two-cell embryo with a dense array of microfilaments formed intensely beneath the plasma membrane of each blastomere (f).

Spermatozoa penetrated into ZP and were found in the cytoplasm of MII oocytes by 6 h after IVF (28/63, 44.4%, Figure 1(a); Table 1). Although a large proportion of these penetrated oocytes were still arrested at MII stage, 2 of the 28 oocytes (7.1%) had already resumed the second meiosis and were in telophase II. Eighteen MII oocytes (18/63, 28.6%) were classified as nonfertilized oocytes as determined by the absence of a spermatozoon within the ooplasm, or spermatozoa were only attached at the ZP of the oocytes. Two of 12 MI oocytes were also penetrated by spermatozoa.

### 3.2. In Vitro Fertilization of Partial Zona-Digested Oocytes

Tyrode's solution dissolved the ZP of buffalo oocytes. After exposure for 45 sec, approximately one-third of ZP was digested. Fertilization rate, in terms of pronuclear formation, of ZP-digested oocytes (60.4%), was not significantly different compared to 54.8% of control (*P* > .05, Table 2).

### 3.3. Redistribution of Cytoskeleton and Chromatin Configurations

Percentage of maturation and fertilization of swamp buffalo oocytes at 12, 18, 24, and 30 h after IVF are shown in Table 3; the maturation rate did not significantly differ among groups (*P* > .05). However, the rate of fertilization at 12 h after IVF was significantly lower than the other time points (*P* < .05). The results demonstrated that the presumptive zygotes showed decondensation of both male and female chromatin by 12 h after IVF. A small proportion of MII oocytes (18/63, 28.6%) underwent activation. Of these activated oocytes, 5 and 13 oocytes were in telophase II (Figure 1(b)) and pronuclear stage, respectively. During decondensation of sperm chromatin, densely stained microtubules were observed and continually elongated to form the sperm aster that radiated from the nucleation site of the sperm centrosome at the base of the decondensing sperm head (Figure 1(c)). It revealed that the developmental rate of the male and female pronuclei was asynchronous at 12 h after IVF (a lack of synchrony between the male and female pronuclei). By 18 h after IVF, 33 of 65 MII oocytes (50.8%) were recorded as being fertilized, and 30 of them had already reached the pronuclear stage. At this time, microtubule networks of sperm aster simultaneously increased in size and extended throughout the ooplasm of the fertilized oocytes.

The percentage of fertilized oocytes at 24 h after IVF was 49.2% (29/59). Most of fertilized oocytes (25/29) were also in pronuclear stage. Apposition of the pronuclei (syngamy) was observed in 14 of these 25 fertilized oocytes. At this stage, microtubules were intensely stained between the two pronuclei (Figure 1(d)). Cleavage was firstly observed by 30 h after IVF (14 of 27 fertilized oocytes). A dense array of microfilaments formed between two presumptive blastomeres (Figure 1(e)) and later formed an intensely labeled layer beneath the plasma membrane of each blastomere after cell cleavage (Figure 1(f)). In addition, the percentage of “nonfertilized” MII oocytes was clearly demonstrated in the present study. Most MII oocytes that failed to progress through the second meiotic division had only a number of spermatozoa bound onto or within the zona pellucida, suggesting the important role of spermatozoa on the failure of fertilization.

## 4. Discussion

The chronology of early embryonic development in terms of cytoskeleton redistribution and chromatin configurations in swamp buffalo embryos during the first cell cycle was examined and firstly described in this study. Until recently, overall success of *in vitro* embryo production in swamp buffalo has remained relatively poor when compared to the results obtained from riverine buffalos and bovines, for example, due to the limitation of fundamental knowledge associated with gamete interaction at fertilization. Oocyte maturation rates in this study (Table 3) were similar to the range of 47 to 85% in other observations [18–20] and fertilization rates of these MII oocytes (ranging from 28.6–50.8%) in terms of oocyte activation and cell cleavage were variable, which is also in accordance with other previous studies [16, 21–24]. While a number of factors have been demonstrated to be involved in the fertilization rate of swamp buffalo oocytes including sperm quality [25], concentration [16], and culture media used [26, 27]; intrinsic factors within the cytoplasm of MII oocytes (as usually referred to as cytoplasmic maturation) also play a critical role during the activation of both male and female gametes during fertilization and early embryo development [28–30]. During IVF, spermatozoa rapidly undergo several modifications including a remodeling of the sperm plasma membrane, and spermatozoa then become hyperactivated [31] and capable of binding to mature oocyte via specific sperm-binding ZP3 receptors [32]. As a consequence, spermatozoa undergo the acrosome reaction and finally penetrate the ZP and fuse with the oolemma [33]. In this study, spermatozoa penetrated through the ZP of mature oocytes by 6 h after IVF which was similar to previous reports in bovines [34, 35]. However, it has been reported that a spermatozoon was already present in the cytoplasm of bovine oocytes as early as 2-3 h after IVF [36]. Many factors were likely involved in the difference in speed of sperm penetration among studies such as type of spermatozoa (fresh or frozen), coincubation time, and capability of spermatozoa to respond to the capacitating medium during IVF [6, 35, 36]. In addition, our study demonstrated that sperm penetration in MI swamp buffalo oocytes was also possible which was similar to reports in bovine [37, 38] and canine [39]. However, it appears that intrinsic factors within the ooplasm of oocyte also play a crucial role in determining the fate of sperm decondensation such as immature oocytes that cannot support postfertilization events of sperm head decondensation [40]. 

This study revealed that a dense network of a thread-like structure of tubulin (referred to as the sperm aster) was formed at the base of decondensing sperm head during gamete activation. The evidence that elongated radial sperm aster was involved in the movement and apposition of male and female pronuclei of buffalo's zygote suggested that the centrosomal material is primarily paternally inherited and is similar to previous reports in other mammalian species such as human [1, 2], sheep [3], rabbit [4], porcine [5], bovine [6–8], and rhesus monkey [9]. However, this is different in mouse because cytoplasmic microtubules in the cytoplasm originate from maternal centrosomes and sperm astral microtubules were not detected in the decondensing paternal chromatin [10, 41].

We found the significant differences in the percentage of oocytes being fertilized between 12 h and the others (*P* < .05). The low numbers of fertilized oocytes presented in Table 3 indicate that a large proportion of penetrated sperms underwent pronuclear formation during 12–18 h after IVF, while formation of female pronucleus in oocytes occurred before the decondensation of sperm head. After decondensation of sperm chromatin, the proportion of pronuclear stage embryos was asynchronously observed by 12–18 h after IVF. Although not exhaustively examined, this asynchronous development of zygotes has been postulated to be caused by several factors such as delayed decondensation of sperm chromatin [35], variation of individual animals [6, 42], and variation of cell cycle transition (M phase to G2 stage) following sperm entry [43, 44]. In this study, the development of buffalo zygotes was more synchronized around 18 h which was similar to a report in bovine [6].

In buffalo, pronuclei movement was clearly influenced by the sperm aster. We found that the male and female pronuclei were positioned at the center of the oocyte, in which the sperm aster mediated by microtubules was concentrated between the two pronuclei until the zygote entered the first mitotic phase and cleaved to the two-cell stage. This result is in agreement with previous report in sheep [3], but in contrast to rabbit and mouse. The sperm aster of the latter species was a transitory structure that dispersed rapidly around the male pronucleus [4, 11]. In addition to the role of microtubules during fertilization and early embryo development, this study also indicated that microfilaments also played an important part in the fertilization and cleavage in swamp buffalo embryos. These filaments were concentrated as a cell furrow predominantly in the middle line of the dividing cell and subsequently located just beneath the plasma membrane as previously reported in Xenopus [45], mouse [46], porcine [12], and bovine [47] embryos. Actin microfilaments were regulated by actin-related protein such as profilins [48] and have also been documented to be actively involved in the redistribution of mitochondria [49, 50], polarization of embryos, and also pronuclear apposition [51].

In our study, we firstly found that fertilization failure was associated with the absence of spermatozoa in the MII ooplasm, even though a number of spermatozoa were tightly bound to the zona pellucida. Although partial digestion of ZP using Tyrode's acid did not affect sperm penetration, this approach also failed to improve fertilization rate when compared with non-Tyrode treated control. It is postulated that other factors such as quality of frozen-thawed sperm [52] and culture environment (low versus high oxygen tension) should be taken into account rather than only assessing the sperm motility prior IVF. Other advance sperm parameters, such as functional membrane integrity, mitochondrial membrane potential, and acrosome integrity, may be additionally required [25].

In summary, this study is the first paper to examine and describe the chronology of swamp buffalo embryo development in terms of redistribution of cytoskeleton and chromatin configurations during the first cell cycle. The study demonstrates that a microtubule organizing center is formed at the area of sperm centrosome and plays an important role in the migration and apposition of pronuclei, whereas actin microfilaments actively involves in cellular cleavage. Fertilization failure of buffalo oocytes, at least in our current culture system, is predominantly caused by poor sperm penetration. However, partial digestion of ZP did not improve fertilization rate in this species. Other factors associated with fertilization failure in buffalo oocytes are needed to be characterized.

## Figures and Tables

**Table 1:** The sperm penetration of swamp buffalo MII oocytes at 6 h after IVF analyzed by confocal laser scanning photomicrography.

No. of oocytes examined	No. of MII oocytes (%*)		Non-MII/Nonfertilized (%*)
	Sperm penetrated	Nonsperm penetrated	
63	28 (44.4)	18 (28.6)	17 (27.0)

*Percentages are expressed in relation to the total number of oocytes used for *in vitro* maturation.

**Table 2:** Percentage of maturation and fertilization of swamp buffalo oocytes treated with Acid Tyrode's solution at 18 h after IVF.

Type of oocytes	No. of oocytes	No. of MII oocytes (%)*	Fertilization rate (%)**
Partial zona digested	64	42 (65.6)	60.4^a^

COCs	43	31 (72.1)	54.8^a^

^a^Within a column, differences were considered to be significant at *P* < .05.

*Percentage is expressed in relation to the total number of oocytes used for *in vitro* maturation.

**Percentage is expressed in relation to the number of MII oocytes.

**Table 3:** Percentage of maturation and fertilization of swamp buffalo oocytes at 12, 18, 24, and 30 h after IVF.

Time after IVF (h)	No. of oocytes (replicates)	No. of MII oocytes (%)*	No. of fertilization (%)**	Embryo development		
				(% of fertilized oocytes)		
				OA (%)	PF (%)	CC (%)
12	103 (9)	63 (61.2)	28.6^a^	5 (27.8)	13 (72.2)	0 (0)
18	97 (7)	65 (67.0)	50.8^b^	3 (9.1)	30 (90.9)	0 (0)
24	91 (7)	59 (64.9)	49.2^b^	4 (13.8)	25 (86.2)	0 (0)
30	87 (8)	56 (64.4)	48.2^b^	4 (14.9)	9 (33.3)	14 (51.8)

^a,b^Within a column, differences were considered to be significant at *P* < .05. OA = oocyte activation; PF = pronuclear formation; CC = cell cleavage.

*Percentage is expressed in relation to the total number of oocytes used for *in vitro* maturation.

**Percentage is expressed in relation to the number of MII oocytes.
